# Effects of milk fermented by *Lactobacillus helveticus *R389 on a murine breast cancer model

**DOI:** 10.1186/bcr1032

**Published:** 2005-04-26

**Authors:** Alejandra de Moreno de LeBlanc, Chantal Matar, Nicole LeBlanc, Gabriela Perdigón

**Affiliations:** 1 Départment de Chimie-Biochimie, Université de Moncton, NB, Canada; 2 Centro de Referencia para Lactobacilos (CERELA-CONICET), Tucumán, Argentina; 3 Cátedra de Inmunología, Facultad de Bioquimíca, Química y Farmacia, Universidad Nacional de Tucumán, Argentina

## Abstract

**Introduction:**

Antitumour activity is one of the health-promoting effects attributed to the lactic acid bacteria and their products of fermentation. Previous studies in mice demonstrated that bioactive compounds released in milk fermented by *Lactobacillus helveticus *R389 contribute to its immunoenhancing and antitumour properties. The aim of the present work was to study the effects of the consumption of milk fermented by *L. helveticus *R389 or its proteolytic-deficient variant, *L. helveticus *L89, on a murine hormone-dependent breast cancer model.

**Methods:**

Mice were fed with milk fermented by *L. helveticus *R389 or *L. helveticus *L89, during 2 or 7 days. The tumour control group received no special feeding. At the end of the feeding period, the mice were challenged by a subcutaneous injection of tumour cells in the mammary gland. Four days post-injection, the mice received fermented milk on a cyclical basis. The rate of tumour development and the cytokines in serum, mammary gland tissue and tumour-isolated cells were monitored. Bcl-2-positive cells in mammary glands and cellular apoptosis in tumour tissue were also studied.

**Results:**

Seven days of cyclical administration of milk fermented by either bacterial strain delayed or stopped the tumour development. Cytokines demonstrated that *L. helveticus *R389 modulated the immune response challenged by the tumour. IL-10 and IL-4 were increased in all the samples from this group. In comparison with the tumour control, all test groups showed a decrease of IL-6, a cytokine involved in oestrogen synthesis. Seven days of cyclical feeding with milk fermented by *L. helveticus *R389 produced an increase in the number of apoptotic cells, compared with all other groups.

**Conclusion:**

This study demonstrated that 7 days of cyclical administration of milk fermented by both strains of *L. helveticus *diminishes tumour growth, stimulating an antitumour immune response. Compounds released during milk fermentation with *L. helveticus *R389 would be implicated in its immunoregulatory capacity on the immune response in mammary glands and tumour, which were correlated with the cytokines found at the systemic level. The milk fermented by *L. helveticus *R389 was able to modulate the relationship between immune and endocrine systems (by IL-6 diminution), which is very important in oestrogen-dependent tumour and induced cellular apoptosis.

## Introduction

Considerable advances have been made in recent years towards an understanding of the molecular factors involved in breast cancer development, but for women in most Western countries breast cancer still remains a major cause of death. There are genetic and environmental factors that increase the chances of breast cancer, and the most common breast cancer types are oestrogen dependent. Some factors, such as diets rich in cultured dairy products, may inhibit the growth of many types of cancer, including breast tumours and the most investigated to date, colon cancer.

Live microbial feed supplements added to beneficially affect the host animal are known as probiotics [1]. Lactic acid bacteria (LAB) are the microorganisms most commonly used as probiotics to favour some biological functions in the host. LAB have been shown to exert effects on the immune system of the consumer and to increase the resistance to neoplasia and infections [2]. Consumption of LAB and milks fermented by them can increase the systemic immune response (macrophage function and number of immunoglobulin-secreting cells) [3,4] as well as increase the local immune responses in the mucosal areas (IgA-positive cells in the intestine, bronchus and mammary glands [5]). For these and other reasons, there is a steady increase in the consumption of fermented dairy products (i.e. yoghurt and other fermented milks) containing viable LAB.

Immunostimulation by fermented milks as a mean of keeping the host immune system in a permanent state of alert has been shown to successfully prevent different cancers [4,6,7]. Beneficial effects of fermented products in colon cancer prevention have been widely reported [8,9]. Studies carried out with an animal model of colon cancer showed inhibition of the tumour through yoghurt feeding, demonstrating that yoghurt modulated the immune system response and exerted its antitumour activity through its anti-inflammatory capacity [10,11]. This effect was observed by long-term cyclic yoghurt consumption, which inhibited promotion and progression of the experimental intestinal tumour [12].

In addition to LAB, fermented milks can possess other nonbacterial components produced during fermentation that contribute to immunogenicity and to other properties like their antitumour activities.

Matar and colleagues [13] have reported different roles and functions of biologically active peptides released from fermented milks. Peptides and free fatty acids released during fermentation were shown to increase the immune response. In this way, peptidic fractions liberated during milk fermentation with *Lactobacillus helveticus *R389 stimulated the immune system and inhibited the growth of an immunodependent fibrosarcoma in a mouse model [14]. The peptidic profiles of milk proteins were significantly different after fermentation by LAB, suggesting that microbial proteolysis could be a potential source of bioactive peptides [15]. Milk fermented with *L. helveticus *R389, a bacterium with high protease and peptidase activity, exerted an antimutagenic effect, while a mutant strain (*L. helveticus *L89) deficient in proteolytic activity did not [16]. In a similar way, milk fermented with the proteolytic strain increased the number of IgA-positive cells in the small intestine as well as in the bronchus of mice, but fermented milk obtained with the proteolytic-deficient mutant strain did not show the same *in vivo *results [6].

Fractions separated by dialysis from yoghurt showed tumour inhibition in *in vivo *murine assays [17]. Biffi and colleagues [18] studied the direct effect of milk fermented by five bacteria species (*Bifidobacterium infantis*, *Bifidobacterium bifidum*, *Bifidobacterium animalis*, *Lactobacillus acidophilus*, and *Lactobacillus paracasei*) on the growth of a breast cancer cell line, and reported that the antiproliferative effect was not related to the presence of bacteria in the fermented milk. This study suggested the potentiality offered by fermented milks as producers of compounds with antiproliferative activity useful in the prevention and therapy of solid tumours like breast cancer.

The aim of the present work was to study the effects of the consumption of milk fermented by *L. helveticus *R389 or its proteolytic deficient variant, *L. helveticus *L89, on a murine hormone-dependent breast cancer model, studying the systemic and local immune responses in the mammary glands and tumours.

## Materials and methods

### Animals and diets

BALB/c mice from Charles River Laboratories (Montreal, QC, Canada), weighing 19–21 g were separated into five experimental groups: the tumour control group, where the mice received an injection with the tumour cells; the P(+) 2d group, where the mice were fed with milk fermented by *L. helveticus *R389, proteolytic variant, for two consecutive days (basal 2 days), were injected with the tumour cells, and were then fed cyclically every 5 days with the fermented milk until day 28; the P(-) 2d group, which was the same as the P(+) 2d group except the mice were fed milk fermented with *L. helveticus *L89, the deficient proteolytic variant, instead of R389; the P(+) 7d group, where the mice were fed with milk fermented by *L. helveticus *R389 for seven consecutive days (basal 7 days), were injected with the tumour cells, and were then fed cyclically every 5 days, with the same fermented milk; and the P(-) 7d group, which was the same as the P(+) 7d group except that the mice were fed milk fermented with *L. helveticus *L89 instead of *L. helveticus *R389.

All groups contained 25–30 mice that were fed with a balanced diet *ad libitum*.

### Milk fermentation

Nonfat, dried, low-heat-grade milk without added vitamins A and D (Dairytown Products Ltd, Sussex, NB, Canada) was rehydrated (12% wt/vol) and autoclaved (115°C for 15 min) to make the inoculums. The prepared milk was inoculated with *L. helveticus *R389 (2% vol/vol) and incubated at 37°C during 17 hours. Yeast extract (0.4%) was added to the milk used to grow *L. helveticus *L89 before autoclaving. This milk was then inoculated with *L. helveticus *L89 (2% vol/vol) and incubated at 37°C for 17 hours. Both fermented milks had a concentration of 1 × 10^9 ^colony-forming units/ml at the end of the fermentation period.

The inoculums were added to rehydrated milk prepared in the same manner (2% vol/vol) in 12% milk or 12% milk plus 0.4% yeast extract to start the milk fermentation.

The extent of milk protein proteolysis was evaluated using the *o*-phthaldialdehyde test [19].

### Tumour induction and the feeding procedure

The ATCC tumoural cell line 4T1 was used to induce breast tumour growth. Each mouse was challenged by a single subcutaneous injection (0.5 ml) of tumour cells (1.4 × 10^4 ^cells/ml) in the upper right mammary gland.

The experimental groups – P(+) 2d, P(-) 2d, P(+) 7d and P(-) 7d – were given a diet supplemented with milks fermented by *L. helveticus *R389 or *L. helveticus *L89 for two or seven consecutive days. At the end of each feeding period the mice were injected with the tumour cells in the same way that the tumour control animals were. Four days after the tumour injection, fermented milks were added again to the diet during two or seven consecutive days (depending on the group), followed by a 5-day break, and then again fermented milk feeding for 2 or 7 days. Feeding was given in this manner cyclically until the end of the experimentation (28 days after tumour induction).

### Obtaining the samples

The following samples were obtained from each group: basal sample (day 0), after 2 or 7 days of fermented milk feeding, and 12, 18, 22 or 28 days after tumour cell inoculation. Mice were anaesthetized intraperitoneally using a mix of ketamine hydrocholoride (Bioniche Animal Health Canada Inc, Ontario, Canada), 100 μg/g body weight, and xylazine hydrochloride (Sigma, St Louis, MO, USA), 5 μg/g body weight. Blood samples were obtained by cardiac punction. For the basal sample, and 12 days after tumour cells injection, mammary glands were removed. In the subsequent samples the tumour was also removed.

To obtain serum, blood was incubated at 37°C during 3 hours and was centrifuged at 1000 × *g *for 10 min. The serums were stored at -20°C until they were used for cytokine measurement.

### ELISA assays of serum samples

To determine the concentration of the different cytokines (tumour necrosis factor alpha [TNF-α], interferon gamma [IFN-γ], IL-10, IL-4 and IL-6) in serum, the BD OptEIA™ mouse cytokine ELISA kits from BD Bioscience (San Diego, CA, USA) were used. The results are expressed as concentration of each cytokine in serum (pg/ml).

### Cytokine-producing cell determination in histological sections

Mammary gland tissue sections (4 μm) from each group were used for immunofluorescence assays. Tissues were prepared for histological evaluation, were fixed in formaldehyde, were dehydrated using a graded series of ethanol and xylene substitute, and were embedded in paraffin.

Cytokines and Bcl-2-positive cells were detected by indirect immunofluorescence following the technique described by de Moreno de LeBlanc and colleagues [11]. Rabbit anti-mouse TNF-α, IFN-γ, IL-10, IL-6 and IL-4 (Peprotech, Inc., Rocky Hill, NJ, USA) polyclonal antibodies (diluted in saponin-PBS) were applied to the sections for 75 min at room temperature (21°C). The sections were then treated with a dilution of goat anti-rabbit antibody conjugated with fluorescein isothiocyanate (Jackson Immuno Research Labs Inc., West Grove, PA, USA).

Bcl-2 protein was measured using the same protocol with a diluted hamster anti-mouse Bcl-2 monoclonal antibody (PharMingen; Becton Dickinson Co., San Diego, CA, USA) and a dilution of the rabbit anti-Syrian hamster antibody conjugated with fluorescein isothiocyanate (Jackson Immuno Research Labs Inc.). The number of fluorescent cells was counted in 30 fields of vision as seen at 1000 × magnification using a fluorescence light microscope. The results were expressed as number of positive cells in 10 fields of vision as seen with 1000 × magnification using a fluorescence light microscope.

### Isolation of mononuclear cells from the breast tumour

Tumours of three mice from each group were removed 18, 22 and 27 days after tumour inoculation and were washed with Hank's balanced saline solution (Sigma) with 4% foetal bovine serum. The cells were separated mechanically and incubated in 0.05% protease/collagenase (Sigma) solution in RPMI 1640 medium (Sigma) with added 10% foetal bovine serum at 37°C and agitated with a magnetic bar for 40 min. The cells collected from supernatant were washed with RPMI 1640 medium. The immune cells were concentrated using a percoll gradient (100% to 55% to 30%), were centrifuged at 800 × *g *for 30 min, and were recovered from the layer between 100% and 55%. Cells were adjusted at 4 × 10^6 ^to 5 × 10^6 ^cells/ml in RPMI 1640 medium. Cell suspensions (20 μl) were placed in each well of an immunofluorescence slide and were fixed with formalin (ICC fixation buffer, PharMingen; Becton Dickinson Biosciences, San Diego, CA, USA).

### Cytokine determination in isolated cells

TNF-α, IL-4, IL-10, IL-6 or IFN-γ were determined in the fixed cells. They were incubated with 1% blocking solution of bovine serum albumin/PBS, were washed with PBS and were incubated with normal goat serum (diluted 1/10). The activity of the endogenous peroxidase was blocked with H_2_O_2_/methanol solution. The cells were then incubated with avidin-blocking and biotin-blocking solutions (avidin/biotin blocking kit; Vector Labs, Inc., Burlingame, CA, USA) to block endogenous avidin and biotin. The cells were incubated with rat anti-mouse TNF-α, IFN-γ, IL-10 or IL-4 polyclonal antibody (diluted in diluent ICC cytokine buffer; PharMingen, Becton Dickinson Biosciences), were washed with PBS, and were incubated with a biotin-conjugated goat anti-rat immunoglobulin-specific polyclonal antibody (PharMingen; Becton Dickinson Biosciences). Biotinylated anti-mouse IL-6 polyclonal antibody (PharMingen; Becton Dickinson Biosciences) was used to determine IL-6-positive cells. Vectastain *Elite *ABC solution (Vector Labs) was added to cells and they were incubated with a DAB kit (Vector Labs). The results were expressed as percentage (number of positive cells in 100 cells counted at 1000 × magnification).

### Apoptosis determination

Apoptosis was evaluated by the presence of DNA breaks, detected in the paraffin cuts using the Apoptosis Detection System kit, Fluorescein (Promega, Madison, WI, USA). The fragmented DNA of apoptotic cells was measured by incorporation of fluorescein-12-dUTP at the 3'-OH ends of DNA using terminal deoxynucleotidyl transferase, which forms a polymeric tail via the principle of the Terminal deoxynucleotidyl Transferase Biotin-dUTP Nick End Labeling (TUNEL) assay. The fluorescein-12-dUTP nick end-labelled DNA was visualized directly by fluorescence microscopy. Cells were defined as apoptotic if the whole nuclear area of the cell was stained fluorescent.

Apoptosis was expressed as the number of apoptotic cells 10 ten fields with 1000 × magnification, using a fluorescence microscope with a standard fluorescent filter.

### Statistical analysis

Comparisons were performed using the software package SigmaStat (SPSS, Chicago, IL, USA).

Comparisons of multiple means were accomplished by one-way analysis of variance followed by a 150 Tukey's post-hoc test. *P *< 0.05 was considered significant. Unless otherwise indicated, all values are the means of three independent trials ± standard deviation.

## Results and discussion

The intestine is the first area of study to assay different properties of probiotics that enter the host by the oral route. This host contains the 'common' mucosal immune system, which ensures that all mucous membranes are furnished with a wide spectrum of secretory antibodies [20]. Both B cells and T cells can migrate from Peyer's patches, found in the small intestine, to mucosal membranes of the respiratory, gastrointestinal and genitourinary tract, as well as to exocrine glands such as the lacrimal glands, salivary glands, mammary glands and prostatic glands [21].

*Lactobacillus casei *CRL 431 administered orally was able to stimulate the IgA cycle, increasing IgA-positive cells not only in the intestine, but also in the bronchus and mammary gland tissues [5].

Previous studies performed in our laboratory showed different effects when mice were fed with milk fermented by *L. helveticus *R389 or its proteolytic deficient variant, *L. helveticus *L89 [6]. Mice fed with the *L. helveticus *R389 showed stimulation of the mucosal immune system. Also, it was observed that 2 and 7 days of feeding with this bacterial strain were optimal to stimulate the mucosal system of mice (data not shown). This strain was also able to prevent the growth of transplantable fibrosarcoma in mice [6]. These previous works led us to study the effect of *L. helveticus *R389 on the growth of breast cancer in an animal model, comparatively with the proteolytic-deficient strain *L. helveticus *L89.

### Tumour growth

**Figure 1:**
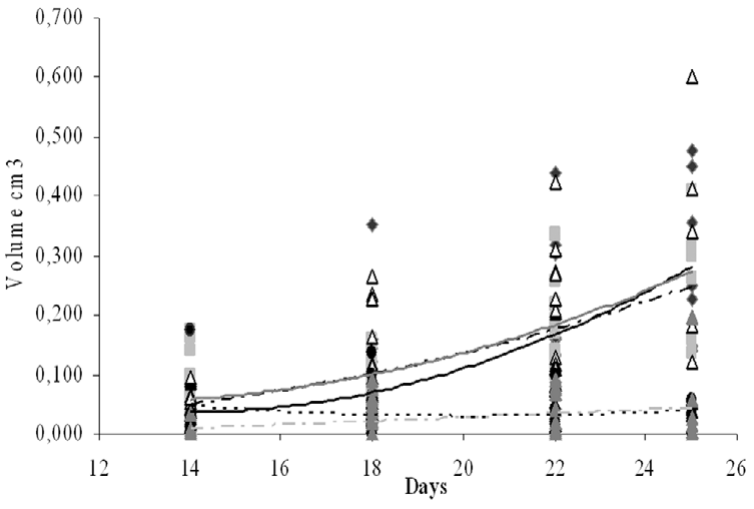
Rate of tumour growth. Results are expressed as the volume (cm^3^) of the tumour for each mouse of different groups (tumour control group, black circle and line; P(+) 2d group, grey square and dark grey line; P(-) 2d group, white triangle and black broken line; P(+) 7d group, black diamond and lines of points; P(-) 7d group, grey triangle and light grey broken lines) with the tendency line for each group.

In the present work, mice were fed cyclically following a previous model of feeding with yoghurt to inhibit a colon tumour in mice, in which yoghurt feeding showed a modulation of the immune response in intestine [11]. Here we showed that mice receiving a 2-day cyclical fermented milk feeding did not show significant differences in tumour volume, compared with the tumour control group (Fig 1). Seven-day cyclical administration of both bacterial strains delayed or stopped tumour development, as compared with the control group (Fig. 1). There were no significant differences between both bacterial strains used in milk fermentation cyclical feeding for either 2 or 7 days (Fig. 1). Either the LAB themselves or some substances released during milk fermentation were responsible for this observed effect: mice fed only with milk (or with milk plus 0.4% yeast extract) did not show differences in the tumour size compared with the control (data not shown)

### Determination of cytokine levels in blood serum

The influence of immune cells in breast cancer development has been reported in different models, but to our knowledge no published reports have studied the *in vivo *immunomodulatory effects of LAB and their relationship with mammary glands or breast cancer. Cytokines have been shown to regulate oestrogen synthesis in breast tumours, stimulating research into these important molecules. In this work, cytokines were assayed in different samples to have a spectrum at a systemic level, and to measure the local response in mammary glands or tumours to study the effect of our fermented milks on the immune response.

**Figure 2:**
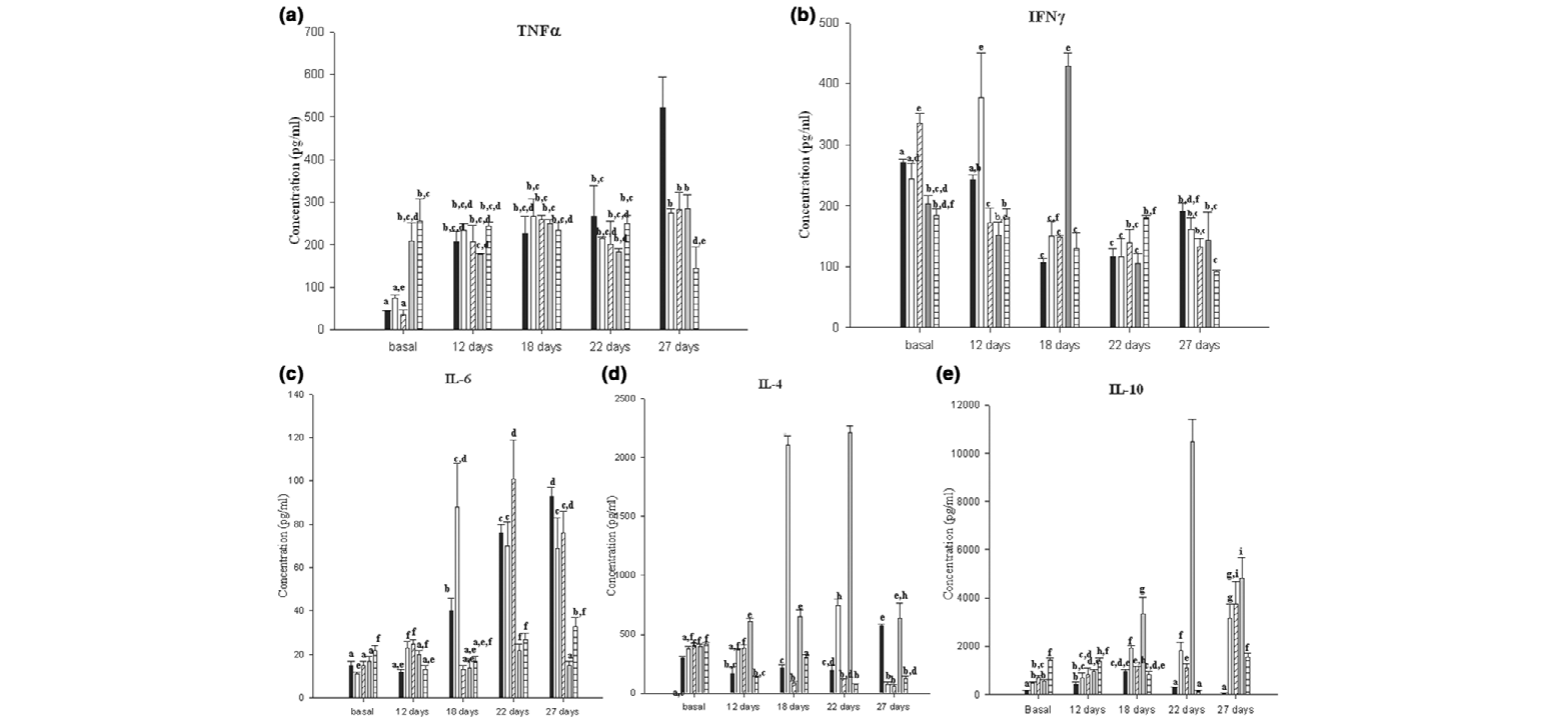
Effect of tumour injection and fermented milk feeding on the serum cytokines. **(a) **Tumour necrosis factor alpha (TNFα), **(b) **interferon gamma (IFNγ), **(c) **IL-6, **(d) **IL-4 and **(e) **IL-10. Results are expressed as the mean concentration of each cytokine (pg/ml) ± standard deviation. Means for each cytokine without a common letter differ significantly (*P *< 0.05).

TNF-α is a cytokine with various functions such as proinflammatory pathway properties, tumour necrosis pathway properties, and apoptosis pathway properties [22,23]. TNF-α levels increased in the serum as a function of time as did the tumour volume in the control group (Fig. 2a). Mice from both 2-day groups showed increases of this cytokine in serum at day 12 (234 ± 14 pg/ml, 206 ± 38 pg/ml and 207 ± 24 pg/ml, for the P(+) 2d, P(-) 2d and tumour control groups, respectively). TNF-α levels remained constant after day 12. Mice that received 7 days of cyclical feeding with milk fermented with *L. helveticus *R389 or *L. helveticus *L89 showed a significant increase (*P *< 0.05) of TNF-α in the basal sample (207 ± 43 pg/ml and 256 ± 51 pg/ml for the P(+) 7d group and the P(-) 7d group, respectively), compared with the tumour control group (42 ± 2 pg/ml). This increase before tumour induction could be related to the decrease in the tumour growth in the mice from these groups. The P(+) 7d group maintained a TNF-α concentration near the basal level throughout the trial, showing a regulation of this cytokine, whereas the P(-) 7d group showed increased TNF-α in the final sample, similar to the control group (530 ± 71 pg/ml and 603 ± 106 pg/ml for 28 days, in the P(-) 7d group and control group, respectively). These results (the TNF-α increase) showed a typical immune response to the tumour [24].

IFN-γ is a cytokine related to the inflammatory response, but it was also reported as a key effector molecule in the immune response against solid cancers. Tumour infiltrating lymphocytes from ovarian tumours released this cytokine upon challenge with MICA-positive tumour cells [25]. In our study, IFN-γ levels varied in the different groups as a function of time (Fig. 2b).

IL-6 is a cytokine implicated in oestrogen synthesis [26], a hormone that the tumour needs for growth. It is also a proangiogenic factor [27], supporting the growth of new blood vessels that are essential for tumour growth. The three groups in which the tumour grew at a faster rate showed elevated levels of IL-6 (Fig. 2c). The P(+) 7d and P(-) 7d groups did not show increased levels of this cytokine throughout the time of the study, suggesting that this IL-6 decrease is involved in one of the mechanisms for the delay of tumour growth.

IL-10 and IL-4 are known as regulatory cytokines, associated with activated Th2 lymphocytes [22]; IL-10 can also be produced by other cell populations such as macrophages and dendritic cells. In different experimental models, TNF-α and IL-10 were demonstrated to have opposite effects [28]. The balance between TNF-α and IL-10 could modulate the effector function of macrophages and cellular apoptosis. IL-4 plays a significant role in controlling both cell growth and modulation of the immune response [29]. This cytokine has antagonist functions to IFN-γ and appears to possess certain anti-inflammatory properties; IL-4 can inhibit the production of several proinflammatory cytokines such as IL-1, IL-6, IL-8, and TNF-α [22]. There was a significant increase (*P *< 0.05) in IL-10 and IL-4 concentrations in the serum obtained from the P(+) 7d group in relation to the tumour control group, beginning at day 18 after tumour injection (Fig. 2e), which could explain the regulation of the immune response observed for TNF-α and IL-6 in these animals. This regulatory response was not observed in the P(-) 7d group. Mice from the P(+) 2d group showed increases for IL-10 concentrations in serum compared with the control, but the levels obtained were significantly lower (*P *< 0.05) than the P(+) 7d group. The P(-) 2d group showed increases of IL-10 compared with the tumour control group, but not compared with the other groups described. These results concur with the development of the tumour observed in these groups of mice.

IL-4 increased in all test groups in the basal sample, in comparison with the control group (18 ± 1 pg/ml). The P(+) 7d group showed the largest increases throughout the study (Fig. 2d).

### Study of cytokine-positive cells in mammary gland tissues

Differences were observed with regard to the systemic levels of cytokines between the groups where the tumour grew and those where it did not; it was possible to observe a regulation of the immune response in the P(+) 7d group, but not in the P(-) 7d group.

The study of cytokine-positive cells in mammary glands allowed an understanding of the local cell response, after mice were fed with fermented milk as well as after tumour injection, in the tumour control group and different test groups.

**Figure 3:**
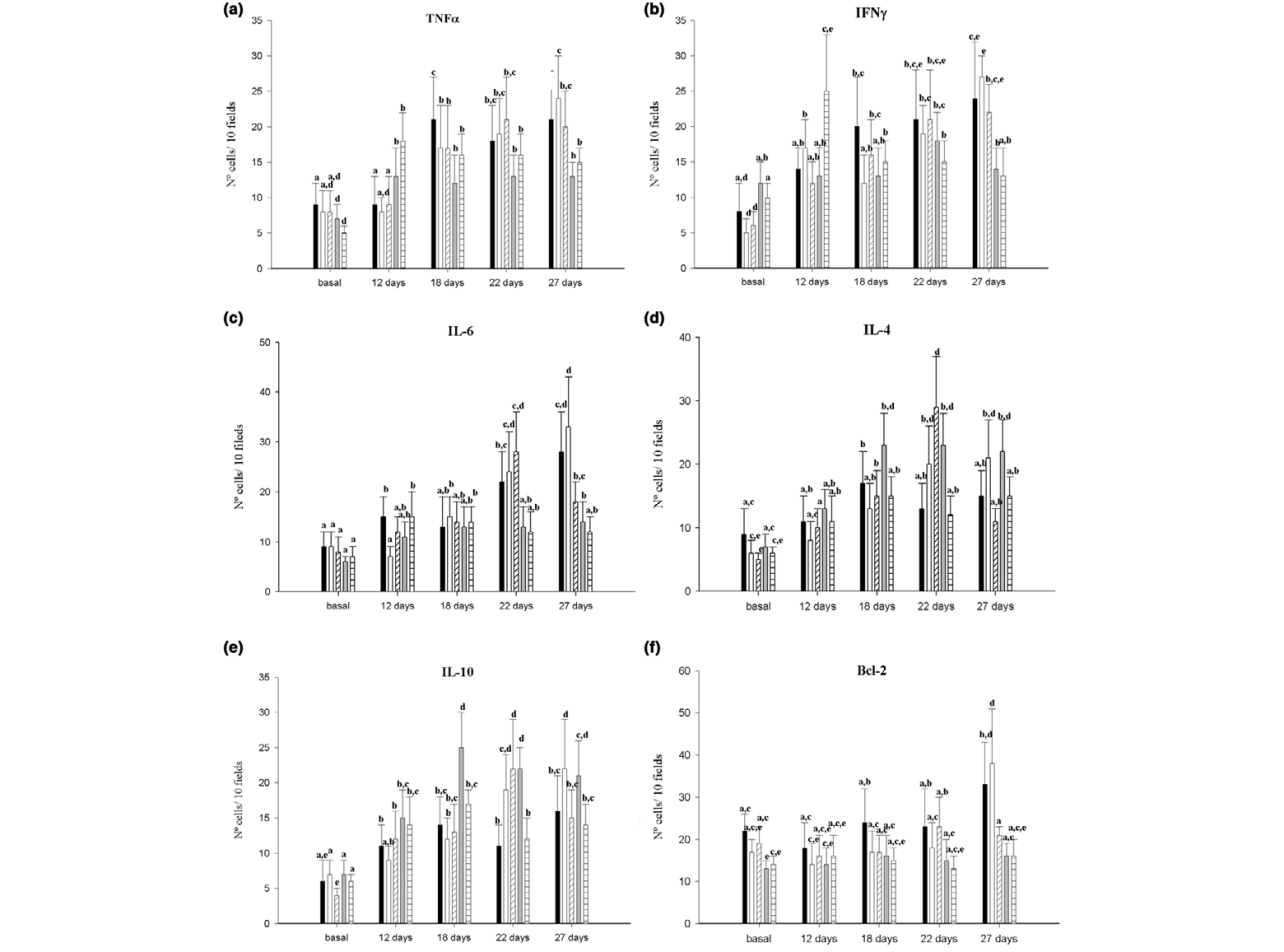
Cytokine-positive cells in mammary glands. Positive cells for each cytokine were counted in histological sections from mammary glands of the tumour control group (black bars), the P(+) 2d group (white bars), the P(-) 2d group (diagonal lined bars), the P(+) 7d group (grey bars) and the P(-)7d group (horizontal lined bars). **(a) **Tumour necrosis factor alpha (TNFα), **(b) **interferon gamma (IFNγ), **(c) **IL-6, **(d) **IL-4, **(e) **IL-10 and **(f) **Bcl-2. Values are means ± standard deviation for *n *= 5. Means for each cytokine without a common letter differ significantly (*P *< 0.05).

TNF-α-positive cells in mammary glands showed very similar patterns to those obtained for this cytokine in serum. TNF-α-positive cells increased in the tumour control group throughout the tumour growth. The same observation was seen in the P(+) 2d and P(-) 2d groups (Fig. 3a). This cytokine increased in the P(+) 7d and P(-) 7d groups 12 days after tumour injection (13 ± 5 cells / 10 fields and 18 ± 5 cells / 10 fields, respectively), and the number of positive cells remained constant afterwards (Fig. 3a).

IFN-γ-positive cells increased in the tumour control group throughout the trial. The P(+) 2d and P(-) 2d groups showed similar values to the control group at the end of the experimental period (27 ± 3 cells / 10 fields, 22 ± 4 cells / 10 fields and 24 ± 5 cells / 10 fields for the P(+) 2d, P(-) 2d and control groups, respectively). Seven days of cyclical feeding, independent of the bacterial strain used, maintained the number of positive cells for this cytokine; however, a significant decrease was observed in the final samples compared with the other groups (14 ± 3 cells / 10 fields and 13 ± 4 cells / 10 fields for the P(+) 7d group and the P(-) 7d group, respectively) (Fig. 3b).

The IL-6-positive cell number was constant and similar in all groups until 18 days after tumour injection. This observation can be explained because this cytokine is related to the synthesis of oestrogen in the mammary gland, a hormone that this tumour cell line needs for proper growth. Eighteen days after tumour cell injection, these cytokine-positive cells increased in the control, P(+) 2d and P(-) 2d groups, whereas both the P(+) 7d and P(-) 7d groups showed no differences in the number of IL-6-positive cells, showing significantly lower numbers in relation to the other groups (Fig. 3c).

IL-10-positive cells increased in the control group after 12 days of tumour injection (11 ± 3 cells / 10 fields) compared with the basal number (6 ± 2 cells / 10 fields), and remained constant throughout the study (Fig. 3e). Mice fed with *L. helveticus *R389 had increased numbers of IL-10-positive cells throughout the time of the entire study, but only the P(+) 7d group showed significantly higher numbers (*P *< 0.05) compared with the tumour control group on days 18 and 22 (25 ± 5 cells / 10 fields and 22 ± 3 cells / 10 fields at 18 days, and 14 ± 6 cells / 10 fields and 11 ± 3 cells / 10 fields at 22 days for the P(+) 7d group and the control group, respectively; see Fig. 3e). This cytokine can be related to the regulation of the other cytokines observed in this group, where increases in TNF-α-positive and IFN-γ-positive cells in mammary glands were observed. This was not the case with the P(-) 7d group, which did not show a significant increase of IL-10-positive cells compared with the tumour control group.

The number of IL-4-positive cells followed the same pattern as the IL-10-positive cells (Fig. 3d): significant increases (*P *< 0.05) were observed in the final samples of the P(+) 2d and P(+) 7d groups (21 ± 5 cells / 10 fields, 22 ± 4 cells / 10 fields and 15 ± 5 cells / 10 fields for the P(+) 2d, P(+) 7d and control groups, respectively) compared with the control.

### Determination of cytokines in tumour-infiltrative cells

Breast tumour tissue contains malignant epithelial cells, stromal cells, adipocytes, lymphocytes and macrophages. The role of tumour-infiltrating immune cells in antitumour immunity, as well as their potential for cancer immunotherapy, has been investigated extensively [30,31]. Lymphocytes and macrophages invade the tumour in response to cytokines such as IL-8 and macrophage chemoattractant protein 1. These lymphocytes and macrophages produce IL-6, IL-6 soluble receptor, and TNF-α. IL-6 is also produced by stromal cells, and TNF-α is also produced by adipocytes. IL-6 is able to stimulate activity of aromatase, an enzyme related to the synthesis of oestrogen from androgens in malignant cells and stromal cells. IL-6 acts through its receptor on malignant cells.

TNF-α showed differences in the isolated cells compared with the other samples (serum and mammary glands). It has previously been reported that cytokines produced by tumour-infiltrating immune cells play an important role in the antitumour response [32]. The positive cells for this cytokine increased in the groups fed with fermented milk where the tumour growth was delayed (the P(+) 7d and P(-) 7d groups), showing that an induction of the production of this cytokine by fermented milk may be playing a biological role in the induction of cellular apoptosis. TNF-α-positive cells increased in the P(+) 7d and P(-) 7d groups 22 days after tumour injection (31 ± 4 cells / 100 and 47 ± 10 cells / 100), but on the last sample (day 27) the number of positive cells did not differ significantly with the control group and the 2-day groups. The same observation was shown with the IFN-γ-positive cells (Table 1).

IL-10 is a regulatory cytokine that can be released by tumour-infiltrating immune cells such as macrophages and lymphocytes. IL-10(+) cell numbers decreased in both the control and the P(+) 2d groups throughout the time of the entire study. The IL-10(+) cells increased significantly (*P *< 0.05) in tumours from P(-) 2d and P(-) 7d groups at 22 days after tumour injection, as compared to the control (18 ± 2 cells / 100, 16 ± 6 cells / 100 and 6 ± 2 cells / 100 for P(-) 2d, P(-) 7d and control, respectively). P(+) 7d was the group with the highest number of IL-10(+) cells (29 ± 7 cells / 100) at 22 days. It is possible to observe another increases of these cells in mice from P(+) 7d group, such as were observed in the other samples (serum and mammary gland tissues), probably to regulate the proinflammatory cytokines (TNF-α and IFN-γ) produced. IL-4-positive cells were variable in all groups, and no significant increases were observed. All mice fed with fermented milk showed decreases in the number of IL-6 (+) cells compared to the tumour control group. This shows a protective effect of the LAB in this oestrogen-dependent tumour, due mainly to the decrease in IL-6. Inter-group differences were observed. After 18 days, P(-) 7d increased these positive cells, and the values were similar to P(+) 2d and P(-) 2d. Only P(+) 7d maintained the number of IL-6 (+) cells significantly lower than the other test groups in all the samples, showing once again the best antitumour response.

### Apoptosis and Bcl-2-positive cell determination

The mechanisms of apoptosis (or programmed cell death) in the inhibition of tumour progression are well documented [33]. Apoptosis is a complex and active cellular process in which individual cells are triggered to undergo self-destruction in a manner that will neither injure neighbouring cells nor elicit an inflammatory reaction. The balance between cell proliferation and cell death is important to maintain equilibrium in different tissues, and a disturbance in this balance may lead to tumour development [34] since the disruption of this type of regulation is a characteristic of tumours.

Considering that cytokines such as TNF-α could be involved in certain apoptotic pathways [23], and that an enhancement of this cytokine was observed in our experimental model, apoptosis induction was studied (Table 2). The P(+) 2d and P(-) 2d groups showed a significant increase (*P *< 0.05) in the number of apoptotic cells at day 18, in relation to the control and other test groups. In mice from the P(+) 7d group, a significant increase was observed in the number of apoptotic cells (*P *< 0.05), compared with all the other groups, beginning at day 22. The increase in cellular apoptosis in the mice of the P(+) 7d group could play a role in the delay of the tumour growth.

Bcl-2 protein is a measure of cell survival due to its anti-apoptotic activity [23], which can be used to stimulate the growth of tumour cells. The increase of cellular apoptosis in mice from the group fed with fermented milk led us to study the Bcl-2 protein.

Significant differences between the groups were not observed when Bcl-2-positive cells were studied in tumour tissues (data not shown), but differences were seen in mammary gland tissues. Bcl-2-positive cells increased significantly (*P *< 0.05) in the final sample for the control group as compared with the basal sample for that group (33 ± 5 cells / 10 fields and 22 ± 4 cells / 10 fields at day 27 and the basal sample, respectively; Fig. 3f). The P(+) 7d and P(-) 7d groups showed significant decreases of Bcl-2-positive cells in mammary glands compared with the tumour control in the same period. These decreases were significant for both groups in the final sample (16 ± 3 cells / 10 fields, 16 ± 4 cells / 10 fields and 33 ± 5 cells / 10 fields for the P(+) 7d, P(-) 7d and control groups, respectively; Fig. 3f) and concur with the apoptosis results.

## Conclusion

Numerous mechanisms in which the immune system plays a role can be involved in the antitumour activity of fermented milks, which could be mediated by the bioactive substances released during fermentation and by the microorganisms used as starter cultures.

Our studies using a model of breast cancer in mice demonstrated that 7 days of cyclical feeding with milk fermented by *L. helveticus *R389 or *L. helveticus *L89 delayed tumour development. This effect was related principally to a decrease in IL-6, a cytokine implicated in the synthesis of oestrogen in both normal and tumour-invaded breast. *L. helveticus *R389, a strain with high proteolytic activity, has been selected for future studies because of its capacity to modulate the immune response. Milk fermented by this LAB induced not only a decrease of IL-6, but also an increase of regulatory cytokines, principally IL-10, and also induced cell apoptosis in the tumour. This observation allows us suggest that substances released in this fermented milk, possibly peptides due to the high proteolytic activity of the bacterial strain, could be related to the regulatory response observed with this fermented milk, which was not observed with the milk fermented by *L. helveticus *L89 (proteolytic-deficient variant).

This is the first report of an *in vivo *study demonstrating the possible mechanisms by which LAB and fermented milks can influence the activity of the infiltrative immune cells in mammary glands, and also to delay or even stop a breast tumour. This study has demonstrated the immunoregulatory capacity of milk fermented by *L. helveticus *R389 on the immune response in mammary glands and tumour, as well as the correlations with the cytokines found at a systemic level. The milk fermented by *L. helveticus *R389 was able to delay tumour growth by its immunoregulatory capacity, and we demonstrated that this fermented milk was able to modulate the relationship between immune and endocrine systems by IL-6 decrease, which is very important in oestrogen-dependent tumours, and by induction of cellular apoptosis.

Research is currently in progress into the antitumour/immunomodulating properties of substances released during milk fermentation by this highly proteolytic LAB, and their effects on breast cancer development.

## Abbreviations

ELISA = enzyme-linked immunosorbent assay; IFN-γ = interferon gamma; IL = interleukin; LAB = lactic acid bacteria; PBS = phosphate-buffered saline; TNF-α = tumour necrosis factor alpha.

## Competing interests

The author(s) declare that they have no competing interests.

## Authors' contributions

AML carried out the study design, animal feeding, data collection (ELISA, immunohistochemistry, immunocytochemistry), statistical analysis, data interpretation, manuscript preparation, and literature search. CM participated in the design of the study, in the data interpretation and manuscript preparation, and in funding the collection. NL participated in the animal feeding, data collection (ELISA, immunohistochemistry, immunocytochemistry), data interpretation, and manuscript preparation. GP participated in the design and coordination of the study, interpretation of data, manuscript preparation, and funding the collection. All authors read and approved the final manuscript.

## Figures and Tables

**Table 1:** Cytokine-positive cells isolated from the tumour

Experimental group	Sample (days)	Cytokine				
		
		Tumour necrosis factor alpha	Interferon gamma	IL-6	IL-4	IL-10
Tumour control	18	24 ± 2^ae^	22 ± 6^ac^	29 ± 2^a^	25 ± 5^a^	18 ± 2^ae^
	22	12 ± 4^bcd^	17 ± 3^ad^	25 ± 3^a^	17 ± 4^ad^	6 ± 2^b^
	27	13 ± 1^bcd^	12 ± 4^b^	23 ± 4^abd^	10 ± 3^ce^	8 ± 2^b^
P(+) 2d	18	21 ± 3^ce^	26 ± 8^cde^	12 ± 3^d^	11 ± 1^bcde^	19 ± 4^a^
	22	9 ± 1^d^	10 ± 2^b^	8 ± 1^cd^	12 ± 2^bcde^	7 ± 3^bc^
	27	11 ± 3^bd^	12 ± 4^bd^	17 ± 3^d^	10 ± 1^ce^	9 ± 2^bc^
P(-) 2d	18	14 ± 6^bcd^	14 ± 5^abd^	10 ± 3^c^	7 ± 2^e^	9 ± 4^bc^
	22	19 ± 4^bce^	13 ± 4^ab^	16 ± 1^bd^	13 ± 5^bcde^	18 ± 6^ae^
	27	12 ± 2^bd^	12 ± 2^b^	17 ± 2^bd^	7 ± 2^e^	8 ± 2^bd^
P(+) 7d	18	22 ± 7^ac^	20 ± 2^ae^	4 ± 2^c^	15 ± 5^dbc^	14 ± 3^ace^
	22	31 ± 4^a^	30 ± 5^c^	9 ± 2^c^	8 ± 1^e^	29 ± 7
	27	13 ± 1^bcd^	17 ± 5^abe^	4 ± 1^c^	6 ± 1^e^	12 ± 2^eb^
P(-) 7d	18	12 ± 2^bcd^	14 ± 1^b^	4 ± 1^c^	12 ± 3^bcde^	13 ± 6^acde^
	22	53 ± 15	44 ± 6	17 ± 3^bd^	12 ± 3^bcde^	16 ± 4^ace^
	27	11 ± 4^bd^	24 ± 5^ac^	13 ± 4^d^	9 ± 1^ce^	9 ± 3^bcd^

Results are expressed as means ± standard deviation of cytokine-positive cells for each 100 counted cells (cells/100). Means for each cytokine without a common letter differ significantly (*P *< 0.05).

**Table 2:** Study of the cellular apoptosis in the tumour tissue

Experimental group	Sample		
	
	18 days	22 days	27 days
Tumour control	8 ± 2^a^	17 ± 5^ab^	13 ± 4^ac^
P(+) 2d	27 ± 6^bd^	38 ± 5^d^	19 ± 6^bc^
P(-) 2d	34 ± 6^d^	10 ± 2^ac^	8 ± 2^a^
P(+) 7d	8 ± 2^a^	57 ± 7^e^	61 ± 10^e^
P(-) 7d	9 ± 2^a^	20 ± 3^bd^	11 ± 3^a^

Results are expressed as the mean ± standard deviation of the number of apoptotic cells counted in 10 fields at 100 × magnification (cells/10 fields). Means for each cytokine (IL-10 or IL-6) without a common letter differ significantly (*P *< 0.05).
